# Close relation of arterial ICC-like cells to the contractile phenotype of vascular smooth muscle cell

**DOI:** 10.1111/j.1582-4934.2007.00066.x

**Published:** 2007-06-24

**Authors:** Vladimír Pucovský, Maksym I Harhun, Oleksandr V Povstyan, Dmitri V Gordienko, Ray F Moss, Thomas B Bolton

**Affiliations:** a Ion Channels and Cell Signalling Centre, Division of Basic Medical Sciences, St. George's, University of London, United Kingdom; b Electron Microscopy and Image Resource Facility, Division of Basic Medical Sciences St. George's, University of London, United Kingdom; c Cell and Metabolic Signalling Research Group, School of Medicine and Dentistry Queen's University Belfast, United Kingdom; d Laboratory of Molecular Pharmacology and Biophysics of Cell Signalling, A.A. Bogomoletz Institute of Physiology, Kiev, Ukraine

**Keywords:** vascular smooth muscle cell, interstitial Cajal-like cell, phenotype, smoothelin, resistance artery

## Abstract

This work aimed to establish the lineage of cells similar to the interstitial cells of Cajal (ICC), the arterial ICC-like (AIL) cells, which have recently been described in resistance arteries, and to study their location in the artery wall. Segments of guinea-pig mesenteric arteries and single AIL cells freshly isolated from them were used. Confocal imaging of immunostained cells or segments and electron microscopy of artery segments were used to test for the presence and cellular localization of selected markers, and to localize AIL cells in intact artery segments. AIL cells were negative for PGP9.5, a neural marker, and for von Willebrand factor (vWF), an endothelial cell marker. They were positive for smooth muscle α-actin and smooth muscle myosin heavy chain (SM-MHC), but expressed only a small amount of smoothelin, a marker of contractile smooth muscle cells (SMC), and of myosin light chain kinase (MLCK), a critical enzyme in the regulation of smooth muscle contraction. Cell isolation in the presence of latrunculin B, an actin polymerization inhibitor, did not cause the disappearance of AIL cells from cell suspension. The fluorescence of basal lamina protein collagen IV was comparable between the AIL cells and the vascular SMCs and the fluorescence of laminin was higher in AIL cells compared to vascular SMCs. Moreover, cells with thin processes were found in the tunica media of small resistance arteries using transmis-sion electron microscopy. The results suggest that AIL cells are immature or phenotypically modulated vascular SMCs constitutively present in resistance arteries.

## Introduction

The vascular smooth muscle cell (VSMC) is regarded as a multifunctional mesenchymal cell [1]. Two principal phenotypes of VSMC were postulated historically: ‘contractile’ and ‘synthetic’[2]. The more recent data suggest that a continuum of cell phenotypes ranging from immature/synthetic to contractile is a more accurate representation of the VSMC differentiation process and the phenotypic heterogeneity resulting from it [3–6]. There are new data which also point towards heterogeneous populations of VSMC coexisting in the vascular wall [6–8].

The smooth muscle cell (SMC) expresses a set of proteins, some of which are restricted to and unique for the SMC lineage. Some proteins of this group, such as smooth muscle α-actin, appear early in the SMC differentiation process, while the expression of others, such as SM-MHC, calponin and smoothelin starts at later stages [4,9]. This temporal pattern of expression makes the individual proteins or their combinations useful as markers of the particular developmental stage of SMC.

Cells with irregular bodies and numerous thin long processes, morphologically similar to the Interstitial Cells of Cajal (ICCs), have recently been described in the vasculature [10–12] and elsewhere outside the gastrointestinal tract [13–20]. The cells found in portal vein were positive for c-kit [10], a marker of ICCs, and spontaneously produced rhythmical calcium transients coupled to membrane depolarisations [21], thus potentially acting as pacemakers in this tissue. However, the cells found in resistance arteries, the arterial ICC-like (AIL) cells, were c-kit negative and were not observed to produce depolarizing currents spontaneously [11]. It was therefore of interest to investigate the cell lineage to which AIL cells belong and where they are localized in the arteries, as a step towards elucidating their role. A preliminary account of this work was published in the form of abstract [22].

## Materials and methods

### Cell preparation

Experiments were carried out on single cells isolated from the 1^st^ to 7^th^ order branches of guinea pig mesenteric artery or on segments of these arteries. Dunkin-Hartley guinea-pigs of either sex weighing 200–850 g were sacrificed by cervical dislocation followed by exsanguination, in accordance with the UK Animals (Scientific Procedures) Act 1986 (Schedule 1). Single cells were obtained by enzymic dissociation [11] and stored at 4°C in physiological saline solution (PSS, composition in mM: NaCl 120, KCl 6, glucose 12, HEPES 10, MgCl_2_ 1.2, CaCl_2_ 1.0, pH set to 7.4 with NaOH) until use.

### Confocal microscopy

The cells were imaged using a Zeiss LSM 510 or a Nikon C1 laser scanning confocal microscope. The excitation beam was produced by an argon (488 nm) or a helium/neon laser (543 or 633 nm), and delivered to the specimen *via* a Zeiss Apochromat 63× oil immersion objective (numerical aperture 1.4) or a Nikon CFI Fluor 60×W objective (numerical aperture 1.0). Emitted fluorescence was captured using either Carl Zeiss LSM 510 or Nikon EZ-C1 software. When the cells were scanned in three dimensions, z-slices were 0.1 μm apart.

### Transmission electron microscopy

Vessel segments were isolated and placed in PSS containing 100 μM nicardipine for 3 hrs, to ensure maximal relaxation. The procedure for their preparation was the same as previously described [11]. The preparations were viewed with a Hitachi 7100 transmission electron microscope at 75 kV and digital images recorded with a Gatan column-mounted CCD camera.

### Immunocytochemistry

Except for smooth muscle α-actin labelling, in which case methanol was used, and laminin and collagen IV labelling where live cells were used, single cells or vessel segments were fixed by 4% paraformaldehyde solution in PSS for 10 or 30 min, respectively, washed with PSS and incubated with PSS containing bovine serum albumin (BSA) and Triton X-100. They were then incubated with primary antibodies in PSS containing BSA overnight at 4°C, washed, and incubated for 2 hrs with secondary antibodies conjugated with fluorescent probes. After removing the unbound secondary antibodies by washing with PSS, the preparations were imaged using the laser scanning confocal microscope.

Antibodies used:

1. PGP9.5: mouse monoclonal (clone 13C4, dilution 1:200, final concentration 1.5 μg/ml);
2. vWF: rabbit polyclonal (1:5000, 2.2 μg/ml);
3. smooth muscle α-actin: mouse monoclonal (1A4, 1:800, 5.6 μg/ml);
4. SM-MHC: mouse monoclonal (HSM-V, 1:200, 50 μg/ml);
5. smoothelin: mouse monoclonal (R4A, 1:50, unknown);
6. MLCK: mouse monoclonal (K36, 1:10,000, 2.1 μg/ml), visualised with Alexa Fluor 488-conjugated chicken anti-mouse antibodies;
7. laminin: rabbit polyclonal (1:200, 3 μg/ml);
8. collagen IV: rabbit polyclonal (1:300, 3.3 μg/ml);

Unless specified otherwise, the preparations labelled with mouse primary antibodies were visualized with Alexa Fluor 633-conjugated goat anti-mouse antibodies, and the ones labelled with rabbit polyclonal antibodies with Alexa Fluor 488-conjugated chicken anti-rabbit antibodies. All the secondary fluorescent antibodies were used at dilution 1:500 (4 μg/ml). F-actin was stained with BODIPY 558/568 phalloidin (5 U/ml, 20 min). Nuclei were stained with SYTO 40 (500 nM, 15 min). PSS contained penicillin (20 U/ml) and streptomycin (20 μg/ml) at all times during immunocytochemical experiments.

### Chemicals

BSA, Dulbecco's Modified Eagle's Medium (D-MEM), paraformaldehyde, methanol, Triton X-100 and the antibodies against smooth muscle α-actin, SM-MHC and MLCK were purchased from Sigma. Antibodies against smoothelin were from Monosan (the Netherlands) and the ones against PGP9.5, vWF, laminin and collagen IV were bought from Abcam (UK). BODIPY 558/568 phalloidin and all the secondary antibodies conjugated with fluorescent dyes were bought from Invitrogen (Molecular Probes). BODIPY 558/568 phalloidin was dissolved in methanol, all the other substances in deionised water.

### Analysis of data

Raw confocal imaging data were processed and analyzed using Zeiss LSM 510 or Nikon EZ-C1 software. An image cutting horizontally through approximately the middle of the cell was selected out of a z-stack of images. Such an image was used to calculate the average pixel fluorescence (APF) as a quantification of the cell's fluorescence signal intensity and compare it between the cells. It was calculated using the formula:


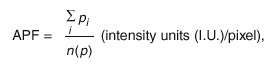
 where *pi* is the intensity of a pixel within the confocal plane of the cell (can be zero) and *n(p)* is the total number of pixels of the plane. This number was calculated by delineating the contour of the cell based on either its fluorescence or, in cases when fluorescence was weak or absent, on transmitted light image taken simultaneously with the fluorescence image, and counting the number of pixels (using software) in such a region. The compared APF was then expressed as a percentage of control APF. Statistical evaluation was carried out and graphs constructed using MicroCal Origin software and final images were produced using CorelDraw 10 software suite.

### Statistics

Data are expressed as mean values ± s.e.m. for the number of cells (n) analysed. Statistical significance was calculated using Student's t-test for paired observations unless other-wise indicated and the differences where *P* < 0.05 were considered significant.

## Results

### Neural and endothelial cell markers

A typical AIL cell is shown in Figure 4A. To test whether the AIL cells are of neural or endothelial origin, single cells adhering to coverslips were immunostained for PGP9.5 (C-terminal ubiquitin hydroxylase), a neural marker [23] or for vWF, a marker of endothelial cells, respectively. The AIL cells did not show positive staining for either of the markers (Fig. 1A and B). In segments of small mesenteric arteries which acted as positive controls, PGP9.5 labelling was intense in fibres in the adventitial layer, although five times lower laser intensity than in the experiment with single cells was used (Fig. 1C). The vWF antibodies labelled granule-like bodies at the level of nuclei in the endothelial cells (Fig. 1D), but not in the smooth muscle layer (not shown). Omitting the primary antibodies resulted in a virtual loss of fluorescence (Fig. 1D, inset).

**4:**
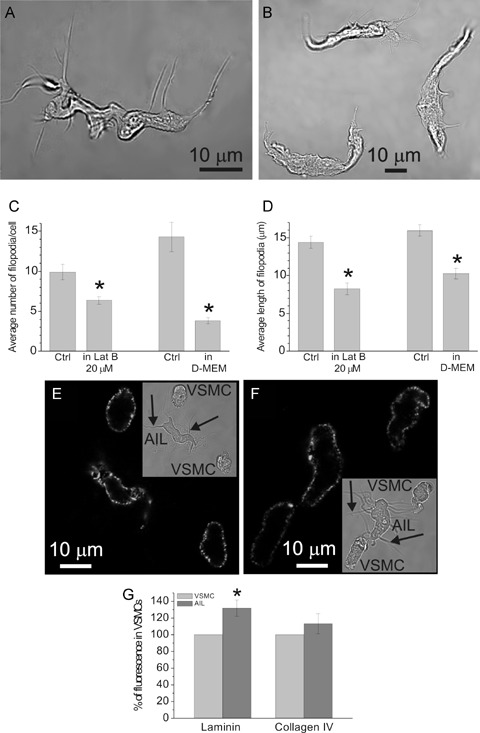
**(A)** Transmitted light image of an AIL cell isolated using the usual procedure. **(B)** Transmitted light image of three AIL cells isolated in the presence of 20 μM latrunculin B. **(C)** The average number of filopodia per cell was decreased from 9.9 ± 1.0 per cell, n = 25 in control (Ctrl) to 6.4 ± 0.5 per cell, n = 39 in latrunculin B (Lat B), P = 0.0006, significantly different. Cell isolation in Dulbecco's Modified Eagle's Medium (D-MEM) also decreased it from 14.3 ± 1.8 per cell, n = 10 in control to 3.8 ± 0.4 per cell, n = 14, P = 0.000002, significantly different. **(D)** The average length of filopodia was decreased from 14.4 ± 0.8 μm, n = 99 in control to 8.2 ± 0.8 μm, n = 55 in latrunculin B, P = 0.000003, significantly different, and from 16.0 ± 0.7 μm, n = 143 in control to 10.3 ± 0.7 μm in D-MEM, n = 53, P = 0.00003, significantly different. **(E)** Image of laminin fluorescence in an AIL cell and two VSMCs obtained by confocal microscopy. Inset shows the same cells in transmitted light. Arrows point to some of the filopodia. **(F)** Same as in **(E)**, but staining for collagen IV is shown. **(G)** Laminin fluorescence of AIL cells was 131.9 ± 9.6% of that in VSMCs (n = 8 cell pairs, P = 0.013, significantly different) and collagen IV fluorescence was 113.3 ± 12.1% of that in VSMCs (n = 9 cell pairs, P = 0.305). Omitting primary antibodies produced virtually no fluorescence in both cases (n = 13 and n = 12, respectively; not shown). * Statistically significant difference to control.

**1:**
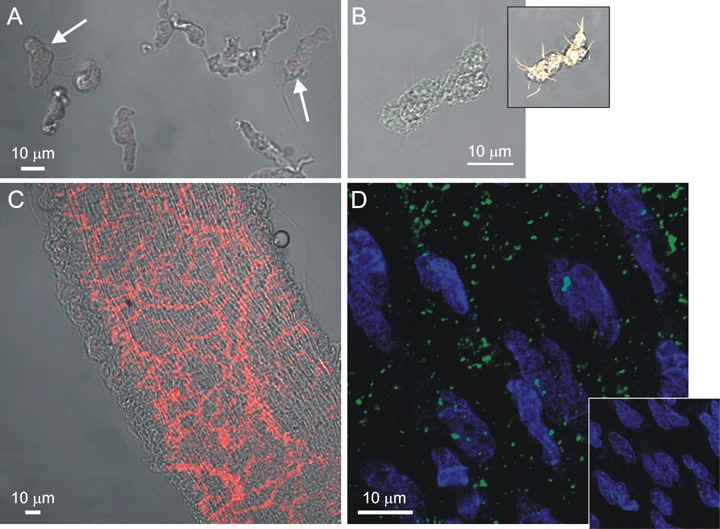
Fluorescent staining for PGP9.5 and vWF. **(A)** A combined image of transmitted light (shades of grey) and PGP9.5 fluorescence (red) of VSMCs and AIL cells (arrows) obtained by confocal microscopy. Neither AIL cells nor VSMCs stained positive for PGP9.5 (n = 24 cells). **(B)** A combined image of transmitted light (shades of grey) and vWF fluorescence (green) of an AIL cell, showing only weak non-specific staining (n = 6). Inset: Same cell stained with BODIPY 558/568 phalloidin (yellow) revealed the presence of otherwise faintly visible filopodia, confirming the identity of an AIL cell. **(C)** Same as in **(A)**, but a segment of mesenteric artery was used, showing the presence of nerve fibers (positive control, n = 5). **(D)** A fluorescent image of an endothelial layer in mesenteric artery segment immunostained for vWF and stained with SYTO 40 for nucleic acids. Nuclei of endothelial cells can be seen in blue. Green colour shows vWF staining of granular appearance, due to its storage in the Weibel-Palade bodies of endothelial cells (n = 4). Inset: As for the main panel, but the primary antibodies to vWF were omitted.

### Smooth muscle cell markers

All the experiments involving SMC markers were carried out by imaging pairs of closely adjacent AIL cells and VSMCs in the same microscope field, to allow for direct comparison between their fluorescent signals.

Our previous work has shown the presence of actin in AIL cells [11], but this was done using a fluorescent derivative of phalloidin, which does not discriminate between the actin isoforms. In this work we tested for the presence of smooth muscle a-actin (SMaA), a smooth muscle specific isoform of actin which is expressed in the early stages of the VSMC development and regarded as a marker of cells with VSMC commitment [4]. Both AIL cells and VSMCs stained positive for SMaA. The fluorescent signal originated from all of the cytoplasm except the central part occupied by the nucleus (Fig. 2B) and, in case of AIL cells, also from filopodia. The signal intensity in AIL cells was significantly lower than that in VSMCs (Fig. 2C).

**2:**
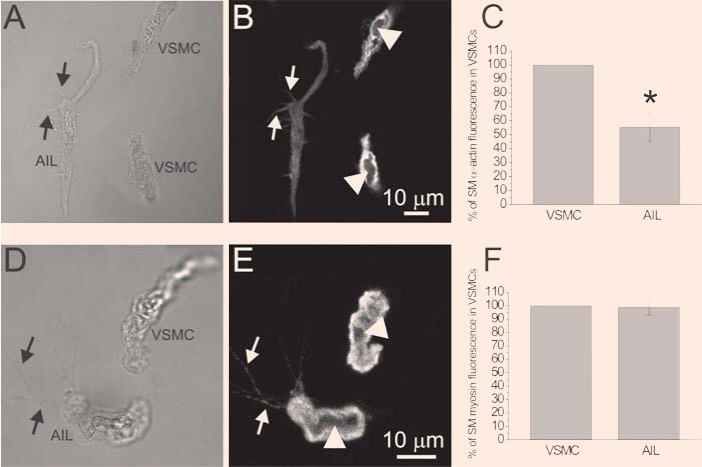
Fluorescent staining for SMA and SM-MHC. **(A)** Transmitted light image of an AIL cell and two VSMCs and **(B)** an image of SMA fluorescence in the same cells, obtained by confocal microscopy. **(C)** SMαA fluorescence of AIL cells was 55.5 ± 10.2% of that in VSMCs (n = 6 cell pairs, P = 0.0074, significantly different). Omitting primary antibodies (n = 9) produced in average 1.8% of the original fluorescence intensity (n = 6; not shown). **(D)** and **(E)** As for **(A)** and **(B)**, but staining for SM-MHC is shown. **(F)** SM-MHC fluorescence of AIL cells was 98.9 ± 5.8% of that in VSMCs (n = 7 cell pairs, P = 0.852). Omitting primary antibodies (n = 10) produced an average of less than 0.1% of the original fluorescence intensity (n = 7; not shown). Arrows point to filopodia of AIL cells. In both cases, fluorescence was found throughout the cytoplasm of VSMCs and AIL cells, except for the centre, occupied by nucleus (arrowheads). * Statistically significant.

SM-MHC is regarded as a more selective VSMC marker than SMA, as it starts being expressed later in the VSMC development, at the stage of immature VSMCs [4]. Its presence in AIL cells and VSMCs has been demonstrated earlier [11]. In this work we compared the SM-MHC signal intensity between AIL cells and VSMCs, which were imaged as pairs of cells (Fig. 2D). The SM-MHC fluorescence signal from the AIL cells was not statistically different from that of VSMCs (Fig. 2F). Weak signal was also detected from the filopodia (Fig. 2E, arrows).

Smoothelin is at present considered to be the most selective marker of contractile VSMCs [4], as it is not expressed in immature forms of VSMC. Imaging of pairs of cells (an AIL cell and a VSMC) has shown smoothelin distribution throughout the cytoplasm of VSMCs, except for the central part, occupied by the nucleus, but significantly less of it (in some cases virtual absence) in the AIL cells (Fig. 3B and C). This result suggested that AIL cells are immature or phenotypically modulated VSMCs.

**3:**
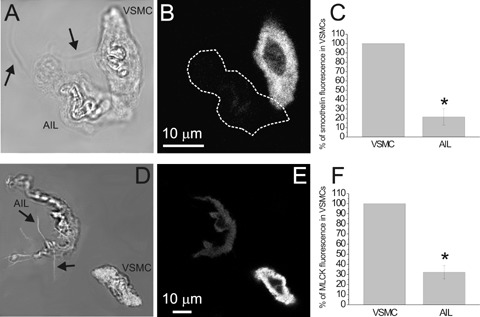
Fluorescent staining for smoothelin and MLCK. **(A)** Transmitted light image of an AIL cell and a VSMC and **(B)** an image of smoothelin fluorescence in the same cells, obtained by confocal microscopy. **(C)** Smoothelin fluorescence of AIL cells was 21.4 ± 8.7% of that in VSMCs (n = 10 cell pairs from three animals, P = 0.00001, statistically significant). Omitting primary antibodies (n = 29 cells from five animals) produced an average of 2.6% of the original fluorescence intensity (n = 10; not shown). **(D)** Transmitted light image of an AIL cell and a VSMC combined with the image of BODIPY phalloidin fluorescence of the bottom plane (in white; to enhance the visibility of filopodia). **(E)** As for **(B)**, but staining for MLCK is shown. **(F)** MLCK fluorescence of AIL cells was 32.1 ± 6.6% of that in VSMCs (n = 9 cell pairs, P = 0.00001, statistically significant). Omitting primary antibodies (n = 7) produced an average of 8.7% of the original fluorescence intensity (n = 9; not shown). The fluorescence of both markers was localized throughout the cytoplasm of VSMCs and AIL cells, except for the nucleus. Arrows point to filopodia of AIL cells. * Statistically significant.

MLCK is a critical enzyme in the regulation of smooth muscle contraction. As AIL cells were shown to be non-contractile [11], but expressing both actin and myosin, it was of interest to establish their relative level of MLCK expression. Similarly to cellular distribution of SMA, SM-MHC and smoothelin, our experiments show a cytoplasm-wide presence of MLCK (Fig. 3E). The intensity of MLCK fluorescent signal was significantly lower in AIL cells than in VSMCs (Fig. 3F), which is in accordance with their inability to contract and corroborates the view that they are of immature VSMC phenotype.

Control experiments were carried out for each marker. Incubation of cells with primary antibodies alone did not produce any fluorescence, and the incubation with secondary antibodies alone resulted in average fluorescence signals ranging from less than 0.1–8.7% of the original intensity (see legends to Fig. 2 and 3 for details).

### Are AIL cells constitutively present or an artefact of cell isolation?

The finding that AIL cells are closely related to the contractile phenotype of VSMC gave rise to a question whether they are indeed constitutively present in intact arteries, or are just an artefact resulting from phenotypic modulation of VSMCs during cell isolation, due to tissue injury. Several experiments were carried out to address this issue.

To establish whether AIL cells originate from arteries of a specific size/diameter or are found throughout the mesenteric artery tree, a parallel dissociation of arteries pooled into six groups according to their branch order (6^th^ group contained arteries from branches of 6 th or higher order) was carried out. AIL cells were observed in arteries of each branch order (not shown).

If some VSMCs started to modulate their pheno-type and grow filopodia after tissue injury, thus giving rise to AIL cells, then blocking actin elongation would prevent this process and result in the absence of AIL cells in cell suspensions. When 20 μM latrunculin B, an inhibitor of actin polymerization (Kd =∼200 nM [24]), was present immediately after tissue excision and throughout the tissue dissociation, AIL cells were still found in suspension (Fig. 4B). However, the number of filopodia per cell (Fig. 4C) and their average length (Fig. 4D) were significantly smaller. Similarly, cell dissociation in D-MEM (free calcium concentration buffered to 50 μM) also resulted in AIL cells having fewer and shorter filopodia (Fig. 4C and D).

Isolation of single cells from tissue involves destroying the extracellular matrix embedding the cells. Enzymes like collagenase or protease, used in our experiments, can thus degrade the basal lamina of VSMCs. The protein components of basal lamina, laminin and collagen IV, exert a contractile phenotype-promoting influence on VSMCs [25, 26] and their removal could induce a phenotypic modulation into a synthetic/immature phenotype. If excessive basal lamina shedding of VSMCs indeed makes them convert to AIL cells, then the layer of laminin and/or collagen IV on the surface of AIL cells is likely to be sparser than on VSMCs. To test this hypothesis, live single cells (*i.e.* fixation and permeabilization steps were omitted) were immunostained for these proteins and their signal intensities were compared between AIL cells and VSMCs. The surface of AIL cells and VSMCs stained positive for both laminin (Fig. 4E) and collagen IV (Fig. 4F). There was no significant difference between the fluorescent signal intensities of collagen IV, but the laminin signal was significantly stronger in AIL cells than in VSMCs (Fig. 4G). In control experiments, omitting primary antibodies resulted in virtual loss of fluorescence. The cells did not stain for fibronectin, collagen I, elastin or β–actin (not shown), ruling out non-specific adherence of antibodies to the cell surface.

### AIL cells in intact resistance arteries

At present there is no selective marker of AIL cells able to discriminate clearly between them and the VSMCs, which precludes their direct visualization and localization in intact arteries. For this reason, transmission electron microscopy (TEM) was used to check for the presence and localization of cells with the morphology of AIL cells in artery segments. In contrast to our previous work [11], segments of mesenteric artery (1^st^ to 7^th^ order branches) were maximally relaxed by 3 hr exposure to 100 μM nicardipine. The TEM images showed the presence of cells with thin processes scattered in the media (Fig. 5). The processes were in close apposition to surrounding cells, however their exact length and contacts with other cells could not be established from a single tissue section. The thickness of these processes was comparable to that of filopodia seen in isolated AIL cells, but their number per cell appeared smaller.

**5:**
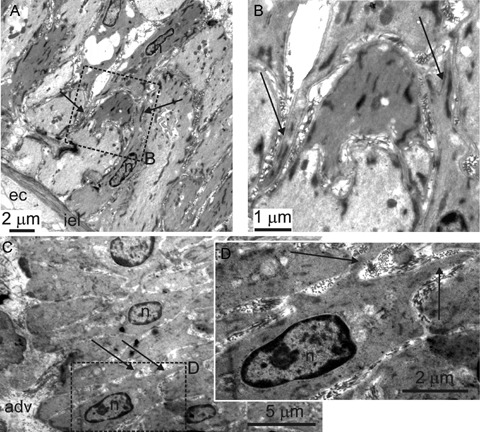
Cells with thin processes in intact mesenteric arteries. Transmission electron micrographs of an oblique section through mesenteric artery. Rectangles in **(A)** and **(C)** delineated by dashed line indicate areas which are shown enlarged in **(B)** and **(D)**, respectively. Adv – adventitia, ec – endothelial cell, iel – internal elastic lamina, n – nucleus. Arrows indicate processes. The cells with thin processes can be seen in the tunica media.

## Discussion

The results of the present work suggest that AIL cells are closely related to the contractile VSMCs and are constitutively present in the resistance arteries of a mammalian species.

Being isolated from arteries, AIL cells could be one of a number of different cell types: neural or endothelial cells, fibroblasts, dendritic cells, myofibroblasts, pericytes, ICCs or immature VSMCs. The results of the present work show that the AIL cells are unlikely to be neural or endothelial cells, as they did not express the markers of these cell types. The results of our previous work [11] have shown that they are c-kit negative and thus unlikely to be the ICCs in the strict sense of word. As they express SMαA, they are not fibroblasts, and the presence of SM-MHC strongly suggests they are not dendritic cells either [27]. The pericytes are only found in pre-capillary arterioles, capillaries and post-capillary venules. The fact that AIL cells were isolated from each order of mesenteric artery branches (external diameter 120–500 μm [11]) makes unlikely the possibility that AIL cells are pericytes.

The presence of SMαA and SM-MHC in AIL cells suggests that they belong to the smooth muscle lineage and are differentiated to at least the level of myofibroblasts. Myofibroblasts are characterized by the presence of fibronectinrich fibronexuses on their surface and a lack of basal lamina [28]. AIL cells were positive for collagen IV and laminin and negative for fibronectin, which indicates the presence of basal lamina and a lack of fibronexuses and strongly suggests they are not myofibroblasts.

Smoothelin is a protein associated with the cytoskeleton and found in contractile SMCs [29, 30]. It has been shown to be an indicator of SMC pheno-type modulation [31]. In small muscular arteries (less than 10 layers of SMCs), more than 95% of SMCs express smoothelin [32]. The very weak or absent smoothelin signal in AIL cells indicates that they are not differentiated to the level of a contractile/mature VSMC. This result and the observation that AIL cells express significantly less MLCK than VSMCs offers one explanation for our earlier observation that AIL cells, despite expressing SMαA and SM-MHC, are unable to contract [11].

Cells identified as arterial ICCs have been described in human aorta and carotid artery [33]. These cells were found along the border between media and adventitia, where they were in direct contact with nerve endings. They too were *c-kit* negative, but a subset of them was positive for neurokinin 1 receptor (NK1R). Preliminary experiments with immunocytochemical staining without the signal amplification did not detect these receptors in AIL cells from guinea-pig mesenteric arteries (unpublished results). At present, there is insufficient data to decide whether there is a relation between arterial ICCs from human conduit arteries and AIL cells from guinea-pig resistance arteries.

Upon enzymatic dissociation of the vessel or injury, the inhibitory influence of the endothelium and of the extracellular matrix on VSMCs is removed and the contractile VSMCs can spontaneously modulate their phenotype into a synthetic one after 3–7 days in cell culture [34–36]. Therefore, the possibility existed that the AIL cells do not occur in intact arteries, but that the freshly isolated VSMCs rapidly modulate their phenotype, turning into AIL cells.

However, several lines of evidence suggest this is unlikely. Firstly, the TEM images of artery segments have shown cells with processes in the tunica media [11]. The thickness of the processes was comparable to that of filopodia seen in isolated AIL cells. In this work, the segments were maximally relaxed to minimize squeezing and folding of cells, which, when observed in tissue sections, can be mistaken for thin processes, a distinguishing feature of AIL cells. However, despite this intervention, cells with thin processes were still observed in artery sections.

Secondly, if VSMCs were indeed modulating their phenotype into AIL cells after the integrity of arteries had been disrupted, it would not be possible to find cells with thin processes in intact arteries, that is before the integrity is disrupted. It was shown that the elongation of filopodia in AIL cells was abolished by latrunculin B, an actin polymerization inhibitor [11]. Continuous presence of supramaximal concentration of latrunculin B during isolation would prevent the development and elongation of filopodia and consequently, no AIL cells would be observed in cell suspension. The most plausible explanation to our observation that AIL cells were present even in the single cell suspension obtained in the presence of latrunculin B is that cells with thin processes were already present in intact arteries before isolation. As the experiments with cell isolation in D-MEM suggest, one reason why the cells in intact tissue have fewer and shorter processes could be the inhibitory influence of an environment rich in nutrients on the growth of filopodia.

Thirdly, if AIL cells were an artefact brought about by shedding of basal lamina from VSMCs and their subsequent phenotypic modulation into an immature/ synthetic phenotype, then the laminin and collagen IV content of AIL cells would be lower than that of VSMCs. Quite the opposite, the experiments have shown that the laminin signal was stronger in AIL cells than in VSMCs and the collagen IV signal not significantly different.

Lastly, smoothelin was all but absent from AIL cells. The smoothelin mRNA levels have been reported to fall sharply by 12 hrs from the removal of tissue, but the protein itself remains present in the cells for up to 5 days [29]. It is therefore unlikely that the VSMCs would be able to down-regulate their smoothelin content within less than 6–7 hrs, which was the longest time from tissue removal to cell fixing.

These observations, together with the immediate presence of AIL cells after isolation and the observed differences in actin cytoskeleton organization between the AIL cells and the VSMCs [11, 37] argue in favour of a resident population of AIL cells within the tunica media. Differences in expression pattern of markers giving rise to heterogeneous subpopulations of VSMCs have been observed both in healthy [38] and in atherosclerotic vessels [39], although such cells were observed in tissue sections and no striking morphological differences between them, like the presence of filopodia, were reported. Recent data [40], showing evidence that in atherosclerosis the VSMCs originate from the local vessel wall and not from progenitor cells in the circulation, further support the idea of resident immature VSMCs. However, the question of VSMC origin is a matter of controversy, as other works suggest existence of a population of circulating, *c-kit* positive VSMC progenitors [41, 42]. ICC-like cells have also been identified in the sections of human and rat atria [43] and ventricles [44] by electron and light microscopy, suggesting the presence of ICC-like cells throughout the cardiovascular system.

Thus, the results suggest that AIL cells are an immature (also referred to as synthetic or epithelioid) phenotype of VSMC, constitutively present in the media of resistance arteries.

We hypothesise that they belong to a VSMC continuum where, on the one extreme lie the contractile, mature VSMCs and on the other extreme the pacemaking, specialized, c-kit positive ICCs (the transition of ICCs into SMCs in the absence of c-kit signalling has been demonstrated in the gastrointestinal tissue [45]). The possibility that the constitutive presence of AIL cells in the vessel wall plays role in vascular remodelling is intriguing, but their migratory and proliferative potential would need to be investigated to address this hypothesis. Together with the cardiac ICC-like cells, which have also been suggested to be uncommitted progenitor cells [43, 44], AIL cells as a possible intermediate VSMC phenotype might become a promising, albeit sparse target for therapeutic interventions. There is insufficient data at present to conclusively establish whether ICC-like cells in arteries are able to act as pacemakers; consequently, their role in vasomotion cannot be ruled out. In practical terms, the constitutive presence of immature VSMCs provides an opportunity to study them on a single cell level in a situation that is as close as experimentally possible to the normal physiological environment.
